# Effect of Electroacupuncture at ST36 on Gastric-Related Neurons in Spinal Dorsal Horn and Nucleus Tractus Solitarius

**DOI:** 10.1155/2013/912898

**Published:** 2013-09-26

**Authors:** Xiaoyu Wang, Hong Shi, Hongyan Shang, Wei He, Shuli Chen, Gerhard Litscher, Ingrid Gaischek, Xianghong Jing

**Affiliations:** ^1^Institute of Acupuncture and Moxibustion, China Academy of Chinese Medical Sciences, 16 Nanxiaojie Street, Dongzhimennei, Beijing 100700, China; ^2^Stronach Research Unit for Complementary and Integrative Laser Medicine, Research Unit of Biomedical Engineering in Anesthesia and Intensive Care Medicine, and TCM Research Center Graz, Medical University of Graz, Auenbruggerplatz 29, 8036 Graz, Austria

## Abstract

The aim of this study was to observe the effect of electroacupuncture (EA) at the ST36 acupoint on the firing rate of gastric-related neurons in the spinal dorsal horn (SDH) and nucleus tractus solitarius (NTS). There were different effects of gastric distention in SDH and NTS in 46 male Sprague-Dawley rats. In 10 excitatory neurons in SDH, most of the neurons were inhibited by homolateral EA. The firing rates decreased significantly (*P* < 0.05) in 10 excitatory gastric-related neurons in NTS; the firing rates of 6 neurons were further excited by homolateral EA, with a significant increase of the firing rates (*P* < 0.05); all inhibitory gastric-related neurons in NTS were excited by EA. The inhibition rate of homolateral EA was significantly increased in comparison with contralateral EA in gastric-related neurons of SDH (*P* < 0.05). There was no significant difference between homolateral and contralateral EA in gastric-related neurons of NTS. EA at ST36 changes the firing rate of gastric-related neurons in SDH and NTS. However, there are some differences in responsive mode in these neurons. The existence of these differences could be one of the physiological foundations of diversity and complexity in EA effects.

## 1. Introduction

Electroacupuncture (EA), a new and modern type of traditional acupuncture, is widely used in treating various types of diseases in a clinical setting with the alterations of peripheral electrical stimulation rather than hand manipulation [1]. Conventional acupuncture or “manual acupuncture” involves the manipulation of the inserted needles by hand, such as lifting, thrusting, twisting, twirling, or other complex combinations. EA is a modification of this technique that stimulates acupoints with electrical current instead of manual manipulations and appears to have more consistently reproducible results in both clinical and research settings [2, 3].

With the excellent pain relief efficacy profile and significant effects in some clinical symptoms, EA is being increasingly accepted by practitioners and patients in the West as well [4, 5]. During the last two decades, a considerable number of studies have investigated the efficacy of EA for the treatment of functional gastrointestinal disorders. Human and animal studies were conducted to explore the effects of EA on gastrointestinal secretion, sensation, motility, myoelectrical activity, and molecular neurobiology [6–8]. The ST36 acupoint (Zusanli), the most important and most frequently used acupoint on the stomach meridian, is considered to be the main point of regulation of gastrointestinal function, promoting gastrointestinal peristalsis and detoxification and protecting the mucosal barrier [9].

The transmission of nociceptive signals can be modulated by powerful controls at the first spinal relays including both the segmental mechanism and systemic mechanism that involve supraspinal structures [10]. It is generally accepted that multiple supraspinal sites of the descending pain modulatory system exert powerful effects on the inhibitory response of acupuncture to the visceral nociceptive messages at the spinal level [11, 12]. It was reported that nociceptive visceral inputs could be inhibited by acupuncture at ST36, and the spinal dorsal horn played a significant role in this process [13]. Many reports suggest that regulation of gastric sensation and motility induced by stimulating ST36 seems to be mediated via vagal reflex in the supraspinal pathway [14–17]. In this study, we focused on these two levels of the central nervous system and observed possible effects of EA at ST36 on the firing rate of gastric-related neurons in SDH and nucleus tractus solitarius (NTS).

## 2. Materials and Methods

### 2.1. Animal Preparations

Experiments were carried out on 46 adult male Sprague Dawley (SD) rats (weight: 250–300 g), which were purchased from the Institute of Laboratory Animal Sciences, China Academy of Chinese Medical Sciences (CACMS), and PUMC (Beijing, China). All manipulations and procedures were carried out in accordance with The Guide for Care and Use of Laboratory Animals issued by USA National Institutes of Health and were approved by the Institutional Animal Care and Use Committee of CACMS. Rats were housed (23 ± 1°C) in groups and maintained under a 12-hour light/dark cycle with food and water available ad libitum. The rats were fasted overnight with free access to water and anesthetized about 4-5 hours with an intraperitoneal injection of urethane (1.0 g/kg, Sigma-Aldrich, St. Louis, MO, USA). All the experiments were done in the daytime; a 2 mm diameter polyurethane tube attached to a 1 cm diameter latex balloon was inserted into the stomach through the small longitudinal incision, which was made in the duodenum about 2-3 cm from the pylorus. A syringe was attached to the cannula to inflate and deflate the balloon. The balloon could be filled with 5 mL air, which is equal to 10 cm H_2_O pressure [18].

### 2.2. Extracellular Recording of SDH and NTS

Twenty-two rats were employed in the experiment of laminectomy, which was performed from the T10 to L1 vertebrae to expose spinal neurons for recording. Extracellular recordings were made with a Tungsten electrode (cusp: 20 *μ*m, impedance: 1.5 MΩ; AM systems, Sequim, WA, USA) which was inserted on the left side of the spinal cord (0.5–1.0 mm lateral to the midline, depth 300–1300 *μ*m). Stability for the recordings was achieved by placing 2% Ringer-agar gel over the surface of the medulla. Wide dynamic range (WDR) neurons of SDH were identified on the basis of characteristic responses to mechanical stimuli applied to the receptive field [19–21]. Extracellular records of WDR were continuously monitored using an MP150 data acquisition system (Biopac, Goleta, CA, USA). Both signals were analyzed offline using the PowerLab data system (PowerLab/4s, ADInstruments, Sydney, Australia) and the Spike 2 package (Cambridge Electronic Devices, Cambridge, UK).

The part of the experiment involving extracellular recording of NTS was performed on 24 rats. The extracellular signals of the NTS neurons (distance to the bregma, AP: −11.3~−14.3 mm; ML: 0~1.3 mm; DV: 4~7 mm) were recorded by glass microelectrodes (10–20 MΩ, pulled by Narishige PE-2 vertical puller from a filamented glass) which were backfilled with 2% pontamine sky blue. Firings of the NTS neurons recorded from the glass electrodes were fed through a microelectrode amplifier (MEZ-8201, Nihon Kohden, Tokyo, Japan). Signals were captured online and analyzed offline using the CED 1401-plus data acquisition system and the Spike 2 package (Cambridge Electronic Devices, Cambridge, UK).

### 2.3. Electroacupuncture

The stimulation electrode was placed at ST36, a hind limb point at which EA or manual acupuncture enhances gastric motility [22]. Based on the descriptions in previous reports [23], the location is on the anterolateral side of the hind limb near the anterior crest of the tibia below the knee under the tibialis anterior muscle. This point was bilaterally stimulated with a 2-3 mA pulse of 0.5 ms duration at a frequency of 20 Hz for 40 seconds by a pair of needle electrodes inserted 3 mm deep into the skin. The electrical current for somatic stimulation was generated by a stimulator (SEN-7203, Nihon Kohden, Tokyo, Japan). 

### 2.4. Experimental Procedure

**Figure 1:**
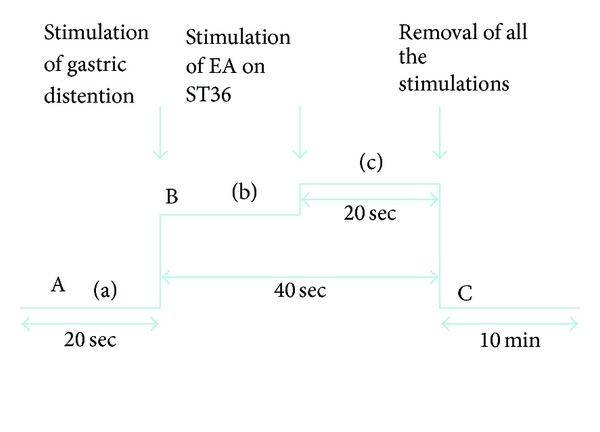
Measurement procedure of the study. A: before stimulation, recording the initial state of neurons (20 sec). B: recording during both kinds of stimulation (40 sec). C: recovery time after stimulation (10 min).

Once the data of the extracellular recorded monitoring had reached a steady state for 5 minutes, gastric distention and EA stimulation were performed. The following measurement periods were analyzed: (a) 20 seconds before the stimulation of gastric distention, (b) 20 seconds during gastric distention stimulation, and (c) 20 seconds stimulation of gastric distention and EA on ST36 (Figure 1). There was a 10-minute recovery time after stimulation. 

### 2.5. Data Analysis

The firing rate and the change rate of spikes in 20 seconds were analyzed. The change rate of spikes is calculated by the number of spikes before stimulation and the number of spikes after stimulation. Data are shown as mean *± *standard error of the mean (SEM). For significance evaluation, data sets with normal distribution were analyzed by paired or unpaired *t* test for two groups or one way ANOVA followed by *q* test, and *P* < 0.05 was considered statistically significant.

## 3. Results

### 3.1. Effects of Gastric Distention in SDH and NTS

**Figure 2:**
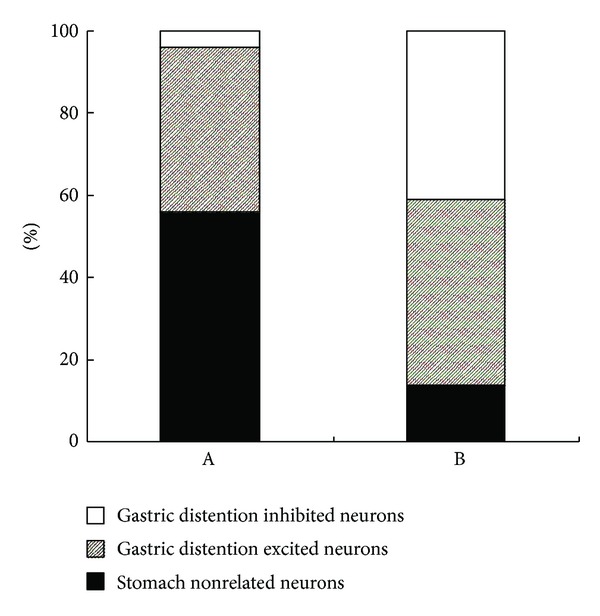
Different effects of gastric distention in SDH and NTS. A: 25 neurons recorded in SDH. There were 14 gastric nonrelated neurons (56%), 10 gastric distention excited neurons (40%), and 1 gastric distention inhibited neuron (4%). B: 22 neurons recorded in NTS. There were 3 gastric nonrelated neurons (14%), 10 gastric distention excited neurons (45%), and 9 gastric distention inhibited neurons (41%).

Of all the 25 neurons recorded in SDH after gastric distention, 11 neurons were sensitized by the stimulation. They showed more than 15% change in the number of spikes and are called gastric-related neurons [15]. Among them, 10 were excited and 1 was inhibited by gastric distention. A total of 22 NTS neurons were recorded in the study; 19 neurons showed apparent excitatory (*n* = 10) or inhibitory (*n* = 9) responses to gastric distention (Figure 2).

### 3.2. Effects of Homolateral EA at ST36 on the Firing Rate of Gastric-Related Neurons in SDH and NTS

**Figure 3:**
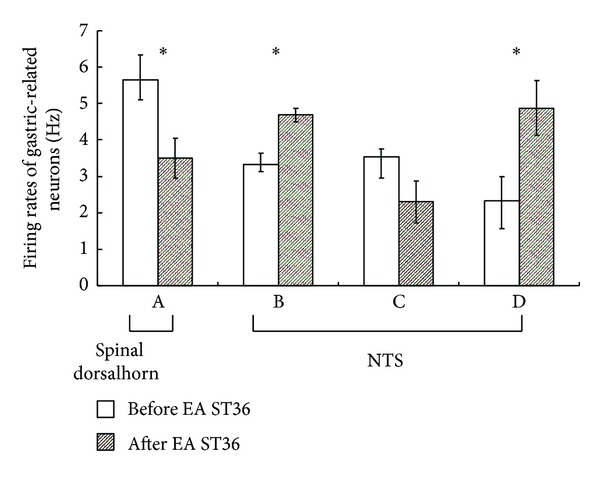
Effects of homolateral EA at ST36 on the firing rate of gastric-related neurons in SDH and NTS. A: stimulation of ST36 in excitatory gastric-related neurons of SDH; the firing rates were inhibited and decreased from 5.65 ± 0.68 Hz to 3.5 ± 0.54 Hz (*P* < 0.05). B: stimulation of ST36 in excitatory gastric-related neurons of NTS; the firing rates increased from 3.32 ± 0.31 Hz to 4.69 ± 0.18 Hz (*P* < 0.05). C: stimulation of ST36 in excitatory gastric-related neurons of NTS: the firing rates increased from 3.53 ± 0.22 Hz to 2.30 ± 0.58 Hz after EA, but there was no significant difference (*n* = 4, *P* > 0.05). D: stimulation of ST36 in inhibitory gastric-related neurons of NTS; the firing rates increased from 2.32 ± 0.67 Hz to 4.87 ± 0.75 Hz (*P* < 0.05).

**Figure 4:**
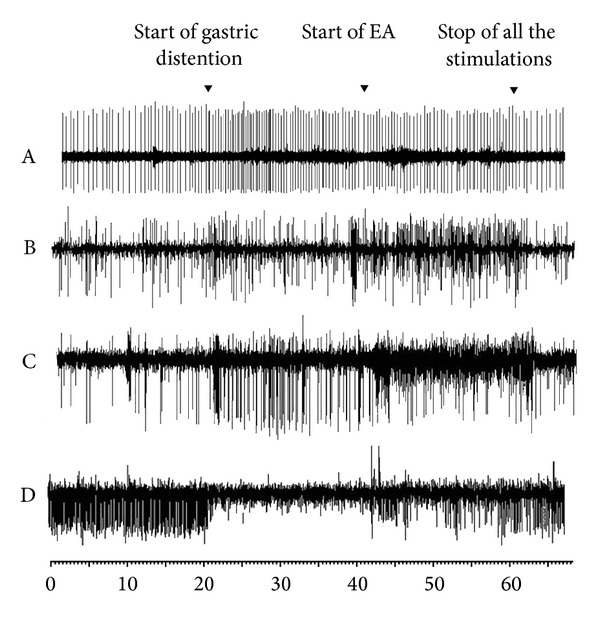
Examples of cells firing in SDH and NTS. A: the change in excitatory gastric-related neurons of SDH caused by EA stimulation of ST36. B and C: the change in excitatory gastric-related neurons of NTS caused by EA stimulation of ST36. D: the change in inhibitory gastric-related neurons of NTS caused by EA stimulation of ST36.

In 10 excitatory gastric-related neurons in SDH, the firing rates of 8 neurons were inhibited by EA. The firing rates decreased from 5.65 ± 0.68 Hz to 3.5 ± 0.54 Hz (*P* < 0.05; A in Figure 3). In 10 excitatory gastric-related neurons in NTS, the firing rates of 6 neurons were further excited by EA, with an increase of the firing rates from 3.32 ± 0.31 Hz to 4.69 ± 0.18 Hz (*P* < 0.05; B in Figure 3). In 4 other excitatory gastric-related neurons in NTS, the firing rates increased from 3.53 ± 0.22 Hz to 2.30 ± 0.58 Hz after EA, but there was no significant difference (C in Figure 3). All the 9 inhibitory gastric-related neurons in NTS were excited by EA. The firing rate increased from 2.32 ± 0.67 Hz to 4.87 ± 0.75 Hz (*P* < 0.05; D in Figure 3) (Figure 4).

### 3.3. Effects of Homolateral and Contralateral EA at ST36 on the Change Rates of Firing Gastric-Related Neurons in SDH and NTS

**Figure 5:**
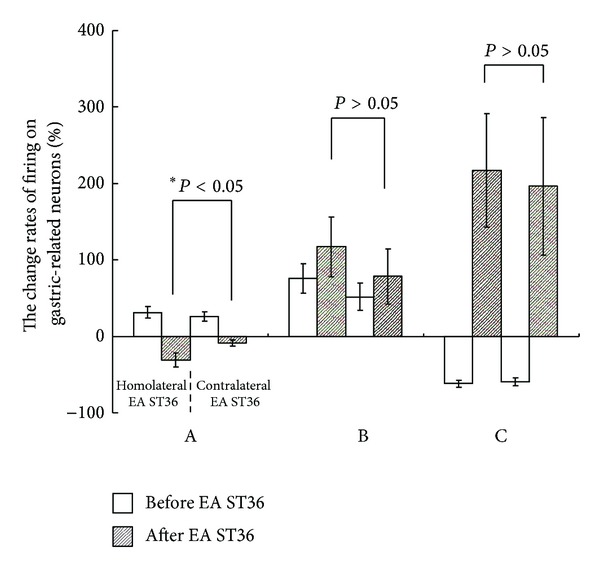
Effects of homolateral and contralateral EA at ST36 on the change rates of firing gastric-related neurons in SDH and NTS. A: the change of firing rate of gastric-related neurons induced by homolateral and contralateral EA at ST36 in SDH. Compared to the contralateral ST36, inhibition rate was significantly increased by homolateral ST36 EA (*P < 0.05). B and C: the change of firing rates of gastric-related neurons caused by homolateral and contralateral EA at ST36 in NTS; there was no significant difference between homolateral and contralateral ST36 (*P* > 0.05).

The inhibition rate induced by homolateral stimulation at ST36 was 30.87 ± 9.06% and significantly increased in comparison with contralateral ST36 stimulation (8 ± 3.59%) in excitatory gastric-related neurons in SDH (*P* < 0.05). There was no significant difference between stimulation of homolateral ST36 (117.03 ± 38.73%) and contralateral ST36 (78.43 ± 36.30%) with regard to change rates of firing excitatory gastric-related neurons in NTS (*P* > 0.05). There was also no significant difference between homolateral ST36 (196.51 ± 89.78%) and contralateral ST36 (217.32 ± 74.44%) with regard to change rates of firing inhibitory gastric-related neurons in NTS (*P* > 0.05) (Figure 5). 

## 4. Discussion

The SDH is the first synaptic relay point for afferent pathways which play an important role in modifying the transmission of noxious input [24]. The integrity of the dorsolateral funiculus is necessary for EA-induced analgesia in the rat tail-flick test [25]. The NTS is a primary center not only for receiving visceral afferents but also for somatic afferents. Commonly labeled medulla oblongata regions were dorsal motor nucleus of vagus nerve (DMV), NTS, and area postrema (AP) following injection of cholera toxin B subunit (CTB) and pseudorabies virus Bartha strain Galactosidase (PRV-Ba-Gal) into the stomach and ST36, respectively [26]. There were some varieties in the response of NTS neurons to gastric distention stimuli and acupuncture at different body surface points [27]. In this study, we observed that NTS neurons presented more diverse responsive modes and sensitivity to gastric distention than did SDH neurons.

A report by Fusumada et al. suggested that the periaqueductal gray (PAG) neurons activated by EA at ST36 might play an important role in the descending pain control system involving gamma aminobutyric acid (GABA), since the PAG has special reference to the SDH and function of pain control [28]. A bilateral microinjection of nociceptin receptor (NOP) antagonist into either the dorsal horn or the intermediolateral column at T1 partially reversed the inhibitory effect of EA at ST36, which suggests that nociceptin in the spinal cord mediates a part of the EA-related modulation of visceral reflex responses [29]. EA at ST36 could extensively regulate the information processing of SDH and induce the modulation of genes/expressed sequence tags (ESTs) in the same direction, which was correlated with neural signal transmission [30]. These results are similar to our study, in which the most popular mode of response to EA was a reduction of the number of spikes in the excited neurons.

EA at ST36 not only regulates gastric activity but also activates neurons in the NTS and DMV significantly [31]. EA at ST36 possibly regulates gastric activity through mediation of the dorsal vagal complex, similar to cardiovascular function, by activating baroreceptor sensitive neurons in the NTS [32]. According to recent evidence, acupuncture at ST 36 can regulate gastric activity, depending on the neural basis or structure and is probably related to the central neurons in the dorsal vagal complex [33]. EA-induced expression of transient receptor potential vanilloid type-1 neuronal nitric oxide synthase (TRPV1-nNOS) and the NTS/gracile nucleus is involved in the signal transduction of EA stimuli via somatosensory afferent-medulla pathways [34]. In our study, the modes of response to EA at ST36 were more complicated in gastric-related neurons of the NTS than in those of the SDH. This is based on the complex components and contact of NTS neurons.

## 5. Conclusion

In summary, EA at ST36 changes the firing rate of gastric-related neurons in the SDH and NTS. However, there are some differences with regard to the responsive mode in these neurons. The existence of these differences could be one of the physiological foundations of diversity and complexity in EA effects.
